# A Cu^II^‐Salicylidene Glycinato Complex for the Selective Fluorometric Detection of Homocysteine over 20 Proteinogenic Amino Acids

**DOI:** 10.1002/open.202200106

**Published:** 2022-06-20

**Authors:** Xuecong Li, Prerna Yadav, Bernhard Spingler, Felix Zelder

**Affiliations:** ^1^ Department of Chemistry University of Zurich Winterthurerstrasse 190 8057 Zurich Switzerland

**Keywords:** amino acids, copper, disassembly approach, fluorometric detection, homocysteine

## Abstract

Homocysteine (Hcy) is a sulfur‐containing α‐amino acid that differs by one methylene (CH_2_) subunit from homologous cysteine (Cys). Elevated levels of Hcy are diagnostic markers of cardiovascular disease and other medical conditions. We present a new Cu^II^‐salicylidene glycinato complex **1** for the selective fluorometric detection of Hcy in water. In the presence of this analyte, the non‐fluorescent copper‐complex demetallates and disassembles into its building blocks. This process liberates a 3‐chloro‐5‐sulfosalicylaldehyde signaling unit and is accompanied by a 51‐fold turn‐on fluorescence at 485 nm (λ_ex_=350 nm). Out of twenty proteinogenic amino acids, only histidine (12‐fold turn‐on fluorescence) and Cys (8‐fold turn‐on fluorescence) trigger some disassembly of probe **1**. In comparison with important pioneering work on the detection of biothiols, this study strikingly demonstrates that structural modifications of chelate core structures steer substrate selectivity of metal‐based probes. Importantly, probe **1** has proven suitable for the detection of Hcy in artificial urine.

## Graphical abstract

**A new Cu^II^‐salicylidene glycinato complex** for the selective fluorometric detection of homocysteine (Hcy) over 20 proteinogenic amino acids in water, following a disassembly approach, is presented. This probe allows for detection of Hcy in artificial urine.

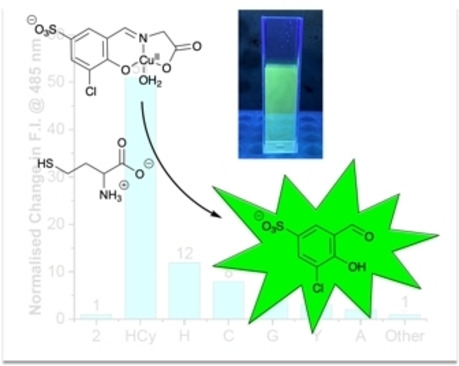

## Introduction

**Figure 1:**
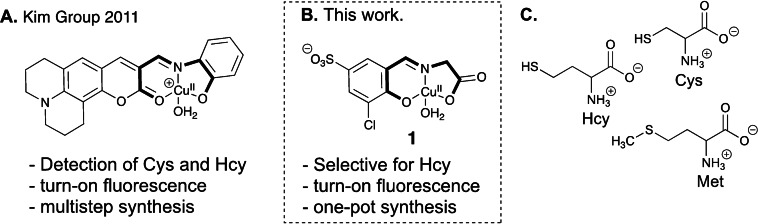
A: Pioneering fluorometric Cu^II^‐iminocoumarin complex for detecting biothiols.^[8]^ B: Cu^II^‐salicylidene glycinato complex **1** for the selective detection of Hcy. The chelate core structures of the tridentate ligands in A and B are shown in bold. C: Sulfur‐containing non‐proteinogenic amino acid (Hcy: homocysteine) and proteinogenic amino acids (Cys: cysteine, Met: methionine).

Homocysteine (Hcy) is a biologically important sulfur‐containing non‐proteinogenic α‐amino acid (Figure 1C).^[1]^ It is enzymatically metabolized into the two proteinogenic amino acids cysteine and methionine (Figure 1C) and represents an intermediate in the recycling of *S*‐adenosylmethionine (SAM).^[2]^ Elevated levels of Hcy are (i) considered as diagnostic markers of cardiovascular disease,^[3]^ acute ischemic stroke,^[4]^ Alzheimer's disease,^[5]^ and other diseases.^[6]^ These anomalies are often caused by a lack of certain vitamin B (i. e., B_12_, B_9_ or B_6_) or a malfunctioning of the corresponding vitamin metabolism.^[7]^

For these reasons, determinations of Hcy are of importance in routine clinical diagnosis. In medicinal laboratories, Hcy is usually detected with chromatographic and mass spectrometric methods or enzymatic assays.^[9]^ Chemical probes for biothiols represent potential cost‐effective alternatives and numerous studies have reported fluorescent chemosensors, chemodosimeters, indicator‐displacement assay and disassembly probes.^[10]^ Despite remarkable progress in this area, discrimination between Hcy and Cys still represents a major challenge.[^10c^, ^11^]

In the context of the detection of biothiols, the development of Cu^II^‐dye complexes has gained considerable attention. In this approach, the paramagnetic metal ion (3d^9^) quenches the fluorescence of the dye by electron transfer into the metal‘s partially filled d‐orbitals. Demetallation of the Cu^II^‐dye ensemble by the biothiol restores the fluorometric response of the dye.[^8^, ^12^] Pioneering examples include a Cu^II^‐iminocoumarin complex for the detection of cysteine (Cys) and Hcy by Kim and coworkers^[8]^ (Figure 1A) as well as a Cu^II^‐iminofluorescein complex for the detection of biothiols by Chen and coworkers.^[12a]^ In these two examples, decomplexation of the Cu^II^‐complexes with the analyte is accompanied by subsequent hydrolysis of the metal‐free imine ligands (i. e., disassembly) that liberates strongly fluorescent signaling units. Despite enormous recent progress in the detection of biothiols, including a Cu^II^‐pyrene complex for two‐photon sensing,^[12b]^ further improvements of selectivity, sensitivity, robustness and water solubility are still desired.

**Scheme 1:**
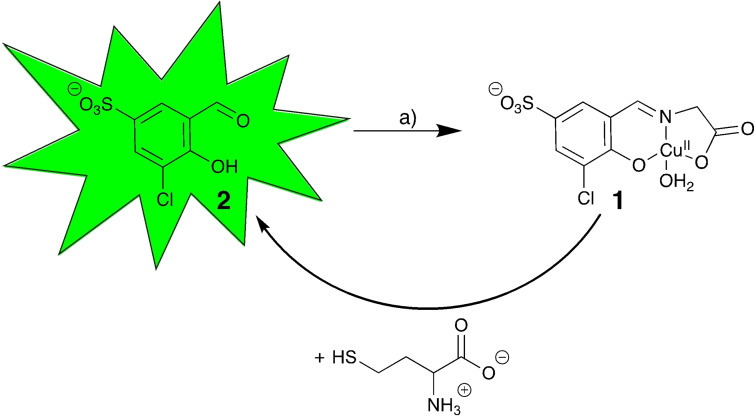
Synthesis and reactivity of probe **1**. a) Glycine, CuCl_2_ ⋅ 2H_2_O

This contribution is motivated by pioneering and recent work with Cu^II^‐dye complexes[^8^, ^12a^, ^13^] and encouraged by our own original studies with Fe^III^‐ and Zn^II^‐salen complexes for (poly)oxophosphate detection.^[14]^ Herein, we report on a Cu^II^‐salicylidene glycinato complex for the fluorometric detection of Hcy over 20 proteinogenic amino acids, including Cys, following a disassembly approach (DA)^[15]^ (Scheme 1). The application for detecting Hcy in artificial urine is demonstrated.

## Results and Discussion

Our reagent, a Cu^II^‐salicylidene glycinato complex is composed of a central Cu^II^‐ion surrounded by a tridentate Schiff base ligand and an aqua ligand (Figure 1B). The Schiff base ligand consists of glycine and a 3‐chloro‐5‐sulfo salicylaldehyde signaling unit (**2**; Scheme 1). The Cu^II^ ion stabilizes and protects the imine functionality against hydrolysis in water (“disassembly”) and quenches the intrinsic fluorescence of the signaling unit **2**.^[20]^ Probe **1** was synthesized in a one‐pot step procedure with signaling unit **2**, glycine and CuCl_2_ ⋅ 2H_2_O in EtOH (Scheme 1 yield: 62 %).

**Figure 2:**
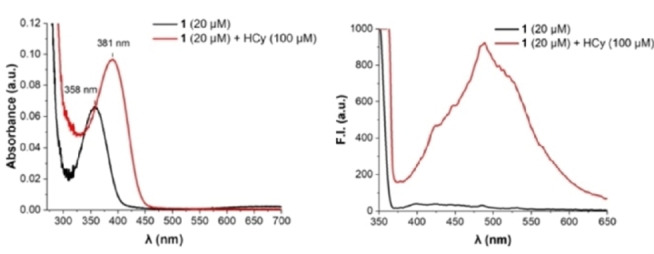
*Left*: UV‐Vis spectra of **1** (20 μM) in H_2_O (pH 7.4, [HEPES]=100 mm) in the absence and presence of Hcy (100 μM) after 30 min incubation. *Right*: Corresponding fluorescence spectra (*λ*_ex_=350 nm).

**Figure 3:**
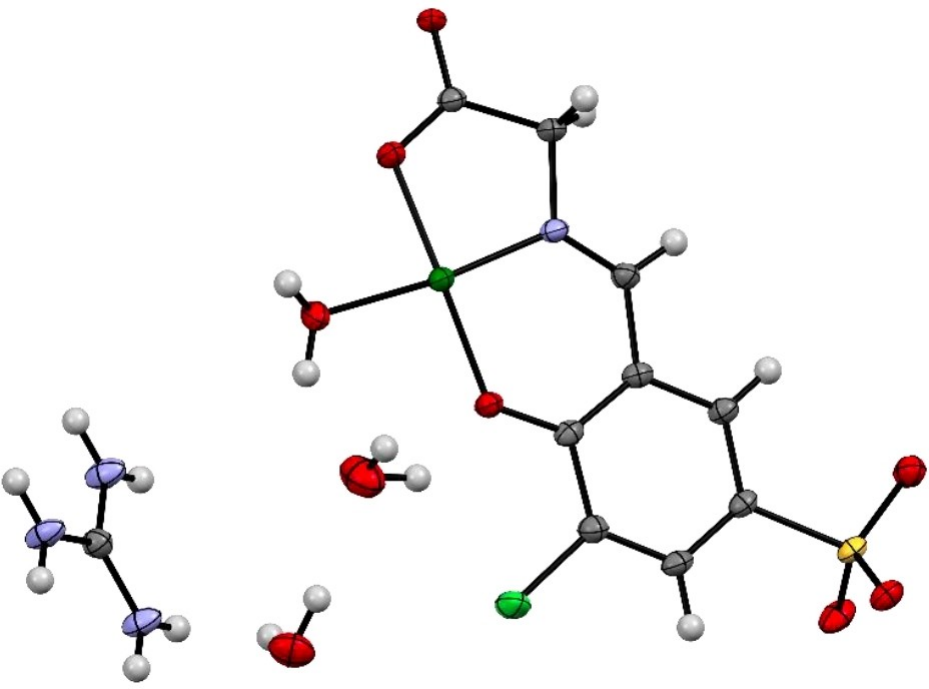
X‐ray crystal structure of the Cu^II^‐salicylidene glycine complex **1**. One out of two molecules in the asymmetric unit is shown. A guanidinium moiety as shown in the figure was used to crystallize complex **1** ⋅ (CH_6_N_3_) ⋅ (H_2_O)_2_. Ellipsoids are drawn at 50 % probability. Colour code: carbon grey, hydrogen white, nitrogen blue, oxygen red, sulfur yellow, chlorine bright green, copper dark green.

Complex formation of the Cu^II^‐salicylidene glycinato complex **1** was accompanied by a blue shift of the absorption maximum of the signalling unit **2** to 358 nm (Figure 2 left, Figure S1) and its strong emission at 485 nm (λ_ex_=350 nm) was quenched in copper‐containing **1** due to ligand‐to‐metal charge transfer (Figure 2 right, Figure S2). The high‐resolution mass spectrum of **1** displays the signal of a [M]^−^ ion at m/z 352.8831 (m/z_calc_ 352.8828) in agreement with the molecular formula of **1** (C_9_H_5_ClCuNO_6_S^−^) (Figure S3). Additional structural proof was obtained from crystal structure analysis (Figure 3, Table S1) depicting a square‐planar copper complex with the tridentate salicylidene glycinato‐, and an additional aqua ligand.

**Figure 4:**
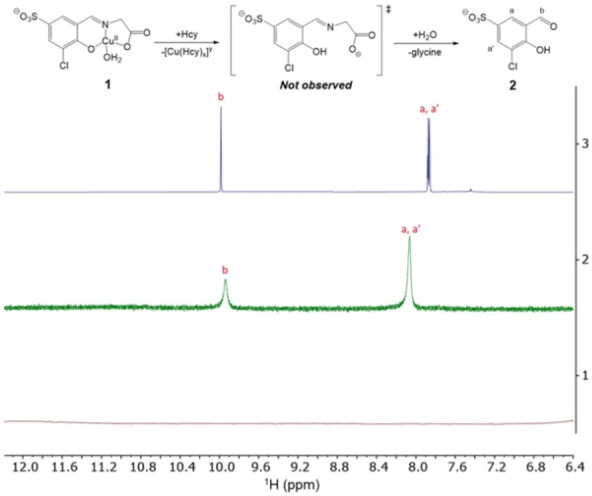
Line 1 (bottom): ^1^H NMR spectrum of **1** (101 mm) in D_2_O.* Line 2 (middle): ^1^H NMR spectrum of **1** (72 mm) in the presence of Hcy (72 mm) after 30 min incubation. Line 3 (top): ^1^H NMR spectrum of **2** (53 mm) in D_2_O. *The ^1^H NMR spectrum of **1** does not show any signal in this region due to the paramagnetism of the Cu^II^‐complex.

Addition of Hcy (100 μm, 5 equiv) to probe **1** (20 μm) led to a redshift of 23 nm to 381 nm in the absorption spectrum (Figure 2, left). This colorimetric change was accompanied by a strong 51‐fold enhanced fluorescence emission at 485 nm (λ_ex_=350 nm) (Figure 2, right). These two characteristic spectrophotometric changes indicate the disassembly of **1** into its subunits and hence, the liberation of the signaling unit **2** in the presence of Hcy (Scheme 1). These observations are supported by ^1^H NMR studies. Whereas the spectrum of paramagnetic **1** (101 mm) did not show any signals in the region between 6 and 12 ppm in D_2_O (Figure 4, line 1), additions of Hcy (1 equiv.; Figure 4, line 2) triggered demetallation of complex **1** and liberation of signaling unit **2** as indicated by the emergence of characteristic protons of the aldehyde and the aromatic moieties of the signaling unit **2** at 9.90 and 8.07 ppm, respectively. From these studies, we did not obtain any evidence for the intermediate formation of the copper‐free salicylidene glycine ligand suggesting that the ligand hydrolyses rapidly upon demetallation in deuterated H_2_O.

**Figure 5:**
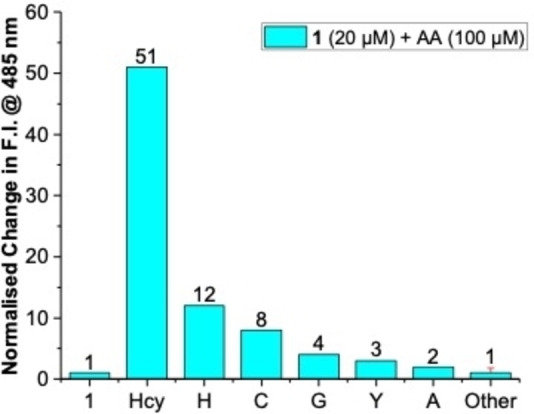
Normalized changes in fluorescence intensity of **1** (20 μm, λ_ex_=350 nm) at 485 nm in the absence and presence of Hcy (100 μm) and 20 common amino acids (100 μM) in H_2_O at pH 7.4 ([HEPES]=100 mm). (**1**: Probe **1**, **Hcy**: Homocysteine, **H**: His/Histidine, **C**: Cys/Cysteine, **G**: Gly/Glycine, **Y**: Tyr/Tyrosine, **A**: Ala/Alanine, **Other**: Other 15 proteinogenic amino acids).^[16]^

The selectivity of probe **1** towards Hcy was tested with 20 proteinogenic amino acids (AAs) including the sulfur‐containing biothiols Cys and Met with emission spectroscopy (Figure 5).

Remarkably, eighteen out of 20 proteinogenic AA (5 equiv.) did not show a significant^[17]^ fluorometric response (Figure 5). In addition to the 51‐fold enhanced fluorescence emission at 485 nm of **1** in the presence of non‐proteinogenic Hcy (5 equiv), only His and Cys (5 equiv. each) led to a 12‐, and 8‐fold enhancement of fluorescence, respectively. These data translate to a 4‐, and 6‐fold selectivity of Hcy over His and Cys, respectively. Both of these interferents contain an additional *N‐* or *S*‐ donor moiety in the side chain suggesting that these functionalities are required for triggering the disassembly reaction of **1**. Overall, the selectivity of **1** is remarkable taking into account that the structurally related tridentate Cu^II^‐iminocoumarin complex (Figure 1A) does not discriminate between Hcy and Cys. The doubly negatively charged chelate core structure of **1** (Figure 1B) is composed of a phenolate, imine and carboxylate subunit and differs in (i) charge, (ii) composition and (iii) donor group with the single negatively charged tridentate ONO chelate core (i. e., lactone carbonyl‐*O*, imine‐*N*, phenolate‐*O*) of the iminocoumarin complex (Figure 1A). The presence of a hard carboxyl functionality instead of a carbonyl‐oxygen imparts additional stability for the hard Cu^II^‐ion and results in a neutral complex‐core structure in **1** in contrast to the positively charged core of the Cu^II^‐iminocoumarin complex (Figure 1A vs 1B). This difference makes demetallation of probe **1** more difficult than of the Cu^II^‐iminocoumarin complex. We suspect that the thermodynamically favored formation of copper‐Hcy complexes or precipitates is the major driving force for the observed selectivity for Hcy over Cys in the disassembly process of **1**. In fact, Hcy has a higher copper(II) stability constant (Cu^II^(Hcy); log β=11.92(1))^[18]^ than all proteinogenic amino acids including Cys.^[19]^ At present, we were not able to isolate or identify copper‐containing reaction products and, hence, a redox‐triggered disassembly process between copper(II) and biothiols to copper(I) and disulfides cannot be excluded.[^18^, ^20^]

Apart from His and Cys, none of the other 18 proteinogenic AA (5 equiv) showed any significant interference with Hcy in a competition assay (Figure S4).

**Figure 6:**
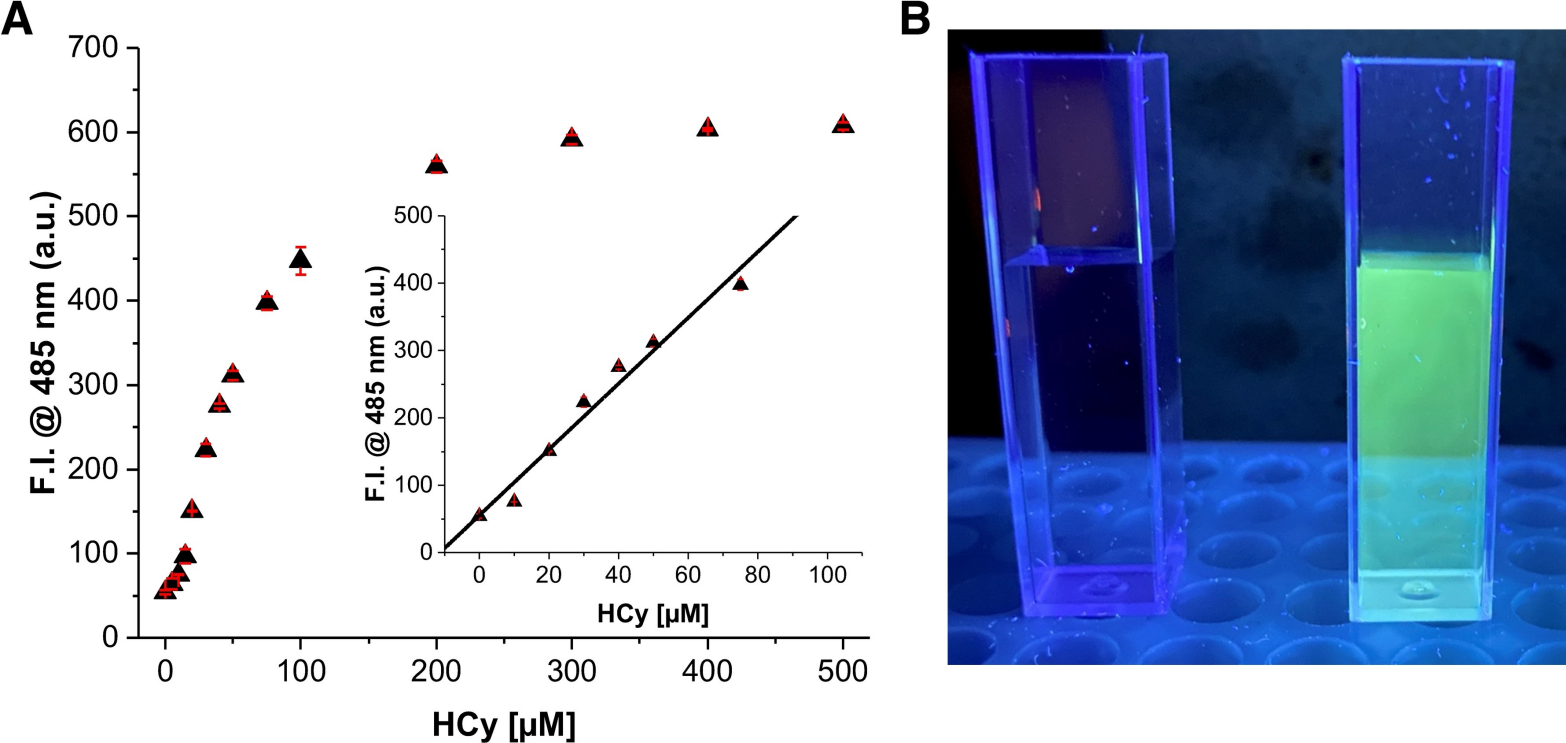
A. Calibration curve ([**1**]**=**20 μm, [Hcy]=0–500 μm; LOD=1.77 μm, R^2^=0.9726) in artificial urine at pH 6.2. B. *Left*: Artificial urine containing **1** (20 μm) in the absence of Hcy. *Right*: Detection of Hcy (20 μm) with **1** (20 μm) in artificial urine using a laboratory UV lamp after 30 min incubation time.

Importantly, with non‐fluorescent copper‐complex **1**, detection of Hcy in artificial urine is possible (Figure 6B). Upon additions of increasing concentrations of Hcy (0–500 μm) to **1** (20 μm) dissolved in artificial urine, fluorescence emission at 485 nm (λ_ex_=350 nm) turns on with a linear range up to 100 μm and a limit of detection (LOD) as low as 1.77 μm (Figure 6).

Despite the strong performance of copper complex **1** in the selective detection of Hcy over 20 proteinogenic AA and its application for detecting Hcy in urine, probe **1** is susceptible for disassembly in the presence of sulfur‐containing glutathione (46‐fold fluorescence enhancement) and hydrogen sulfide (31‐fold fluorescence enhancement) (Figure S5). Although these two biomolecules are usually not encountered in urine, these interferences limit potential applications of **1** for detecting Hcy in living cells.

## Conclusion

In this publication we report on a square planar Cu^II^‐salicylidene glycinato complex **1** for the selective detection of Hcy over 20 proteinogenic AA. In a proof‐of‐principle study, probe **1** was successfully applied for the fluorometric detection of Hcy in artificial urine with a LOD of 1.77 μm. The sulfur‐containing non‐proteinogenic Hcy demetallates probe **1** that subsequently hydrolyses (i. e., disassembly) into its molecular subunits. The liberation of the 3‐chloro‐5‐sulfosalicylaldehyde signaling unit leads to a 51‐fold enhanced fluorescence emission. Little interference is observed from most proteinogenic AA and most remarkably, probe **1** shows a 6‐fold discrimination of Hcy over homologous Cys. In comparison with important earlier work on less selective Cu^II^‐based probes for biothiols, this study demonstrates strikingly that modification of the chelate core structure steers selectivity. We speculate that this effect is not limited to the discrimination of Hcy over homologous Cys with probe **1** and suggest future optimizations of chelate core structures in other metal‐based probes for improved sensing performance. In contrast to proteinogenic AA, discrimination between Hcy and hydrogen sulfide (31‐fold turn‐on fluorescence) or glutathione (46‐fold turn‐on fluorescence) is not possible with probe **1**. This drawback limits applications of the copper‐complex for detecting endogenous Hcy in biological samples and has to be considered in future probe design.

## Supporting Information

Deposition Number 2160859 (for **1**) contains the supplementary crystallographic data for this paper. These data are provided free of charge by the joint Cambridge Crystallographic Data Centre and Fachinformationszentrum Karlsruhe Access Structures service.

## Conflict of interest

The authors declare no conflict of interest.

1

## Supporting information

As a service to our authors and readers, this journal provides supporting information supplied by the authors. Such materials are peer reviewed and may be re‐organized for online delivery, but are not copy‐edited or typeset. Technical support issues arising from supporting information (other than missing files) should be addressed to the authors.

Supporting Information

## Data Availability

The data that support the findings of this study are available in the supplementary material of this article.
