# Supplementary material for: A CuII‐Salicylidene Glycinato Complex for the Selective Fluorometric Detection of Homocysteine over 20 Proteinogenic Amino Acids
Source: ChemistryOpen. 2022 Jun 20;11(6):e202200106. doi: 10.1002/open.202200106 (PMC9208288; doi:10.1002/open.202200106)
Supplement: Supplementary file 1 — Supporting Information [file OPEN-11-e202200106-s001.pdf]

ChemistryOpen

Supporting Information

A Cu^{II}-Salicylidene Glycinato Complex for the Selective Fluorometric Detection of Homocysteine over 20 Proteinogenic Amino Acids

Xuecong Li, Prerna Yadav, Bernhard Spingler, and Felix Zelder*

Table of Contents

S1. Materials and Methods……………………………………………………... S2

S2. Experimental Procedures……………………………………………………… S5

S3. Spectra, Figures and Tables ……………………………………………... S6

S4. References…………………………………………………………………... S11

S1. Material and Methods

General. All chemicals were of reagent grade quality or better, commercially purchased from *Sigma–Aldrich*, *ACROS Organic, Roth, Merck, Fluorochem* or *Fluka* and used without further purification. Signaling unit 3-chloro-5-sulfosalicylaldehyde was prepared as described elsewhere.[S1]Amino acids used in analytical experiments are listed in Table S2. All solvents were of analytical or HPLC grade and were purchased from *ACROS Organic, Roth, Sigma-Aldrich or Fluka*. Milli-Q water was used for all experiments.

Instrumentation.

Centrifugation was performed on an *Eppendorf Centrifuge 5702*.

pH was measured on a *Metrohm* (827 pH Lab) pH-meter.

Masses were balanced on a *KERN ABJ-NM/ABS-N* (d=0.1 mg).

UV-Vis spectra were recorded between 270 and 700 nm at 0.1 nm resolution and 10 points/s on an *Analytik Jena Specord 250 Plus*; λ_{max} (*log* Ɛ) in nm.

FT-IR spectra were measured on a *SpectrumTwo FT-IR Spectrometer* (*Perkin–Elmer*) equipped with a *Specac Golden Gate^{TM} ATR* (attenuated total reflection) accessory; applied as neat samples; $1/\lambda$ in cm^{-1}.

All NMR spectra were recorded on a *Bruker AV2-400* (400 MHz); δ in ppm rel. in ppm rel. to TMS (δ 0.00), J in Hz.

High-resolution electrospray-ionization mass spectrometry (HR-ESI-MS): *QExactive* (*Thermo Fisher Scientific*, Bremen, Germany) with a heated electrospray ionization (ESI) source connected to a *Dionex Ultimate 3000 UHPLC* system.

The crystal of **1·$(CH_6N_3)\cdot(H_2O)_2$** was obtained by a cation screening procedure (E. Slyshkina, F. Kradolfer, B. Spingler, manuscript in preparation), similar to the published anion screening.[S2,S3]

Crystallographic data were collected at 160.0(1) K on a Rigaku-Oxford Diffraction XtaLAB Synergy-S dual source diffractometer. This is a kappa-axis four-circle goniometer with a Dectris Pilatus3 R 200K HPC (Hybrid Photon Counting) detector and Cu and Mo PhotonJet microfocus X-ray sources. Suitable crystals were covered with oil (Infineum V8512, formerly

known as Paratone N), placed on a nylon loop that is mounted on a CrystalCap Magnetic™ pin (Hampton Research) and immediately transferred to the diffractometer. The program suite *CrysAlis^Pro^* was used for data collection, numerical and multi-scan absorption correction as well as data reduction.[S4] The structures were solved with the dual-space algorithm using *SHELXT*[S5] and was refined by full-matrix least-squares methods on F^2 with *SHELXL-2018*[S6] using the *Olex2* GUI.[S7] The graphical output was produced with the help of the program *Mercury*.[S8] CCDC 2160859 contains the supplementary crystallographic data for this paper. These data are provided free of charge by The Cambridge Crystallographic Data Centre *via* www.ccdc.cam.ac.uk/structures.

Fluorescence spectra were recorded on a *Perkin Elmer LS 50B*. The slit-widths for the fluorescence experiments were kept at 10 nm (excitation) and 10 nm (emission) and the excitation wavelength was set at 350 nm for all fluorescence experiments.

Preparation of Artificial Urine

Artificial urine was prepared in Milli-Q water (pH 6.2) containing sodium sulfate (11.97 mM), uric acid (1.49 mM), sodium citrate (2.45 mM), creatinine (7.79 mM), urea (249.75 mM), potassium chloride (30.95 mM), sodium chloride (30.05 mM), calcium chloride (1.66 mM), ammonium chloride (23.67 mM), potassium oxalate (0.19 mM), magnesium sulfate (4.39 mM), sodium dihydrogen phosphate (18.67 mM) and disodium hydrogen phosphate (4.67 mM) according to literature.[S9]

Calculation of the Selectivity Factor

The selectivity factor of complex **1** towards Hcy over other analytes is calculated as follows:

Selectivity factor = $(\mathrm{F.I.}_{\mathbf{1}+\mathrm{Hcy}} - \mathrm{F.I.}_{\mathbf{1}}) / (\mathrm{F.I.}_{\mathbf{1}+\mathrm{other\ analytes}} - \mathrm{F.I.}_{\mathbf{1}})$

$\mathrm{F.I.}_{\mathbf{1}}$ = Fluorescence Intensity of **1** (20 μM).

$\mathrm{F.I.}_{\mathbf{1}+\mathrm{Hcy}}$ = Fluorescence Intensity of **1** in the presence of Hcy (100 μM).

$\mathrm{F.I.}_{\mathbf{1}+\mathrm{other\ analytes}}$ = Fluorescence Intensity of **1** in the presence of other analytes (100 μM).

Calculation of Limit of detection (LOD)[S10]

The limit of detection was determined from the calibration curve from titrations of **1** (20 μM) with increasing concentrations of Hcy (0 - 500 μM) in artificial urine at pH 6.2 using the following equation:

LOD = 3.3×SD/S

SD = Standard deviation of the blank solution (SD = 3.62)

S = Slope of the calibration curve (S = 6.74)

S2. Experimental Procedures

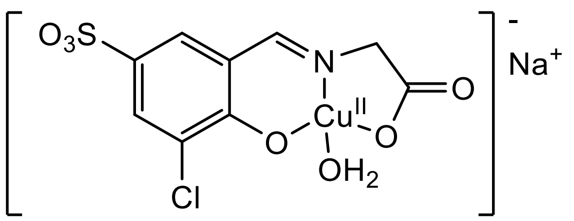

Cu^{II}-salicylidene glycinato complex. 3-chloro-5-sulfosalicylaldehyde (**2**; 60.30 mg, 233 µmol), glycine (17.40 mg, 233 µmol) and an aq. soln. of NaOH (1 M) were stirred in EtOH (20 mL) at 80 °C for 30 min. $CuCl_2 \cdot 2H_2O$ (41.50 mg, 243 µmol) was added to the above mixture and was stirred at 50 °C. The reaction was monitored by UV-Vis spectroscopy. After 30 min, disappearance of the absorbance band of **2** at 378 nm was observed suggesting completion of the reaction. The reaction mixture was cooled to 23 °C and the solvent was removed under reduced pressure to afford a blue solid. The crude product was dissolved in H_2O (1 mL) in a centrifuge tube (50 mL) and was sonicated for 3 min. Acetone (30 mL) was added to the tube which was vortexed for 20 s and then centrifuged. The liquid phase was collected. The solvent of the liquid phase was removed under reduced pressure and the obtained blue solid was dissolved in H_2O (2 mL). The blue solid was frozen in liquid nitrogen immediately and was lyophilized to afford **1** (51.90 mg, 140 µmol) as sodium salt in 62 % yield as a blue powder.

UV-Vis (H_2O, pH 7.39, [HEPES buffer] = 100 mM) (Figure S1): λ_{max} 358 nm (3.54).

HR-MS (H_2O) (Figure S3): *m/z* 352.8831 ($C_9H_5ClCuNO_6S^-$; $[M]^-$; calc. 352.8828).

Crystal Structure of **1**: Figure 3, Table S1.

S3. Spectra, Figures and Tables

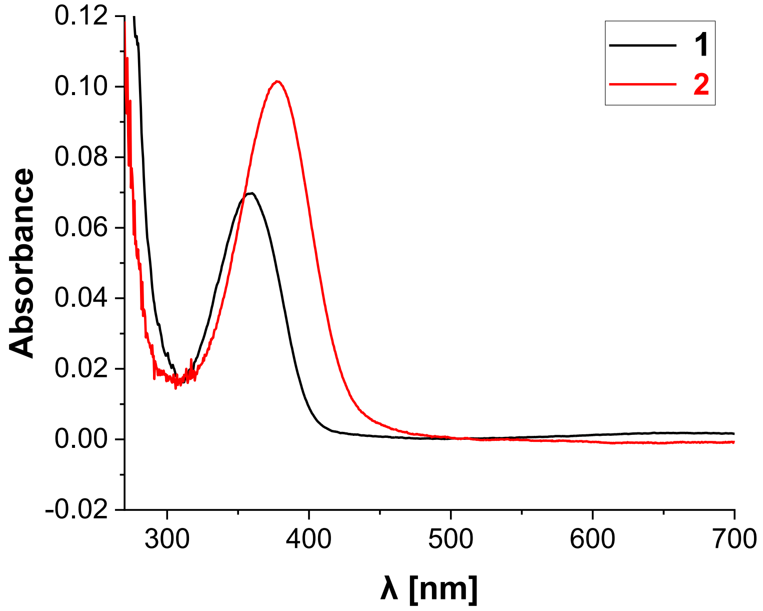

Figure S1. UV-Vis spectra of compound **2** (20 μM) and **1** (20 μM) in H_2O (pH 7.4, ([HEPES] = 100 mM).

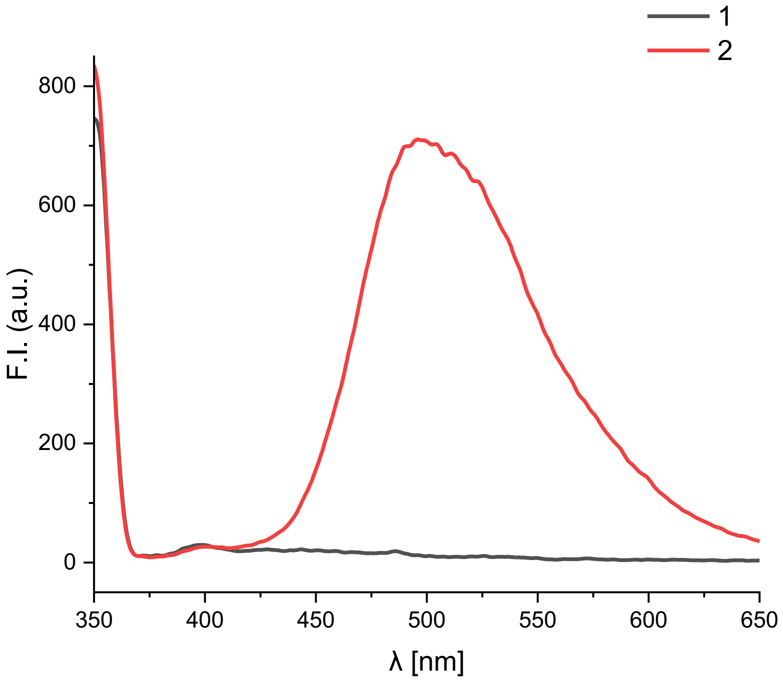

Figure S2: Emission spectra (λ_{ex} = 350 nm) of compound **2** (20 μM) and **1** (20 μM) in H_2O (pH 7.4, [HEPES buffer] = 100 mM).

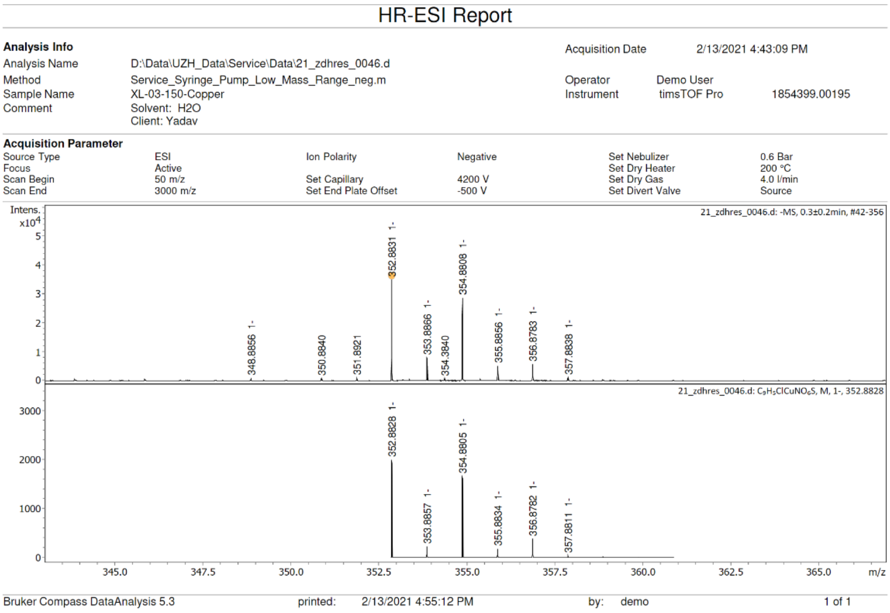

Figure S3: HR-ESI-MS spectrum of compound **1** in H_2O.

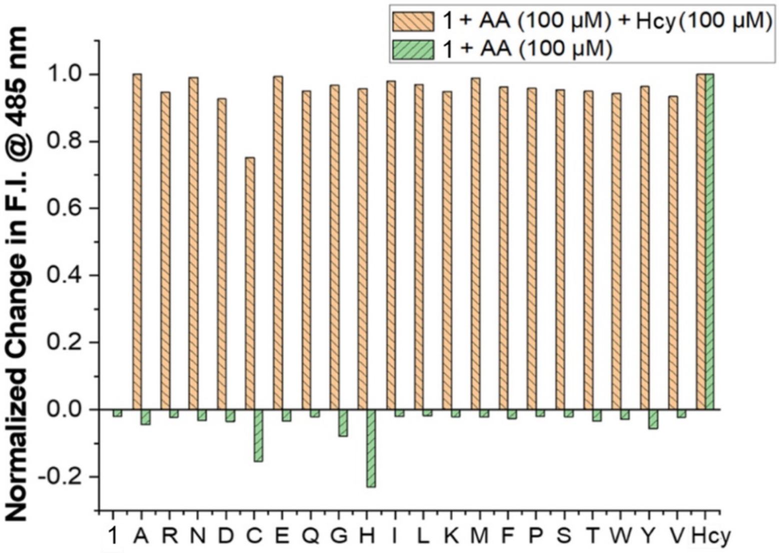

Figure S4. Normalized changes in fluorescence intensity of **1** (20 µM, λ_{ex} = 350 nm) at 485 nm with 20 proteinogenic amino acids (100 µM; Table S2) and with or without the presence of Hcy (100 µM) at pH 7.4 ([HEPES buffer]= 100 mM) (**1**: Probe **1**, **A**: Alanine, **R**: Arginine, **N**: Asparagine, **D**: Aspartic acid, **C**: Cysteine, **E**: Glutamic acid, **Q**: Glutamine, **G**: Glycine, **H**: Histidine, **I**: Isoleucine, **L**: Leucine, **K**: Lysine, **M**: Methionine, **F**: Phenylalanine, **P**: Proline, **S**: Serine, **T**: Threonine, **W**: Tryptophan, **Y**: Tyrosine, **V**: Valine, **Hcy**: Homocysteine.

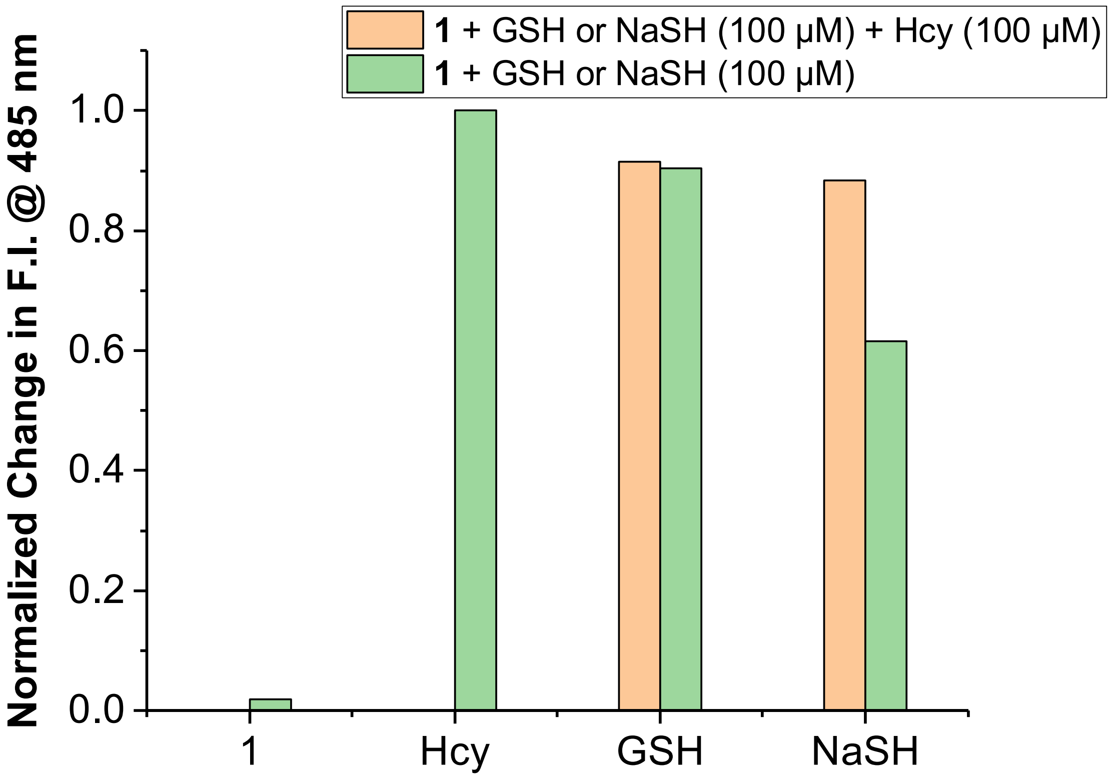

Figure S5. Normalized changes in fluorescence intensity of **1** (20 µM, λ_{ex} = 350 nm) at 485 nm with GSH and NaSH (100 µM) with or without presence of Hcy (100 µM) at pH 7.4 ([HEPES] = 100 mM) (**GSH**: glutathione[S11]).

Table S1: Crystal data and structure refinement for **1·(CH_6N_3)·$(H_2O)_2$**.

Empirical formula	$C_{10}H_{17}ClCuN_4O_9S$
Formula weight	468.32
Crystal system	Triclinic
Space group	P-1
a [Å]	10.88515(10)
b [Å]	11.24758(13)
c [Å]	14.62583(15)
α [°]	97.1691(9)
β [°]	100.1144(8)
γ [°]	100.7476(9)
Volume [$Å^3$]	1708.78(3)
Z	4
Density (calculated) [Mg/m^3]	1.820
Temperature [K]	160.00(10)
Wavelength [Å]	1.54184
Absorption coefficient [mm^{-1}]	4.920
F(000)	956
Crystal size [mm^3]	0.16 x 0.05 x 0.02
Crystal description	blue needle
Theta range for data collection [°]	3.111 to 79.590
Index ranges	-13<=h<=13, -14<=k<=14, -18<=l<=18
Reflections collected	61688
Independent reflections	6898 [R(int) = 0.0437]
Reflections observed	6508
Criterion for observation	I > 2 σ (I)
Completeness to theta	95.0 % to 67.684°
Absorption correction	Gaussian
Max. and min. transmission	1.000 and 0.564
Data / restraints / parameters	6898 / 4 / 490
Goodness-of-fit F^2	1.079
Final R indices [I > 2 σ (I)]	R1 = 0.0335, wR2 = 0.0870
R indices (all data)	R1 = 0.0361, wR2 = 0.0929
Largest diff. peak and hole [e.$Å^{-3}$]	0.660 and -0.526

Table S2: List of stereochemical information and form of amino acids used for analysis.

Amino Acid	Used Form	Molecular Formula
Alanine	DL-Alanine	$C_3H_7NO_2$
Arginine	L-Arginine·HCl	$C_6H_{14}N_4O_2 \cdot HCl$
Asparagine	L-Asparagine	$C_4H_8N_2O_3$
Aspartic acid	DL-Aspartic acid	$C_4H_7NO_4$
Cysteine	L-Cysteine	$C_3H_7NO_2S$
Glutamic acid	L-Glutamic acid	$C_5H_9NO_4$
Glutamine	L-Glutamine	$C_5H_{10}N_2O_3$
Glycine	Glycine	$C_2H_5NO_2$
Histidine	L-Histidine·HCl	$C_6H_9N_3O_2 \cdot HCl$
Isoleucine	L-Isoleucine	$C_6H_{13}NO_2$
Leucine	L-Leucine	$C_6H_{13}NO_2$
Lysine	L-Lysine·HCl	$C_6H_{14}N_2O_2 \cdot HCl$
Methionine	L-Methionine	$C_5H_{11}NO_2S$
Phenylalanine	DL-Phenylalanine	$C_9H_{11}NO_2$
Proline	L-Proline	$C_5H_9NO_2$
Serine	DL-Serine	$C_3H_7NO_3$
Threonine	DL-Threonine	$C_4H_9NO_3$
Tryptophan	DL-Tryptophan	$C_{11}H_{12}N_2O_2$
Tyrosine	L-Tyrosine	$C_9H_{11}NO_3$
Valine	DL-Valine	$C_5H_{11}NO_2$
Homocysteine	DL-Homocysteine	$C_4H_9NO_2S$

S4. References

S1. Berry, K. J.; Moya, F.; Murray, K. S.; van den Bergen, A. M. B.; West, B. O., Water-soluble cobalt(II) complexes of NN′-substituted bis(salicylaldimine-5-sulphonic acids). Oxygen-carrying properties and conversion into cobalt(III) organometallic compounds. *J. Chem. Soc., Dalton Trans.* **1982,** (1), 109-116.
S2. Nievergelt, P. P.; Babor, M.; Čejka, J.; Spingler, B., A high throughput screening method for the nano-crystallization of salts of organic cations. *Chem. Sci.* **2018,** *9* (15), 3716-3722.
S3. Alvarez, R.; Nievergelt, P. P.; Slyshkina, E.; Müller, P.; Alberto, R.; Spingler, B., Single crystal growth of water-soluble metal complexes with the help of the nano-crystallization method. *Dalton Trans.* **2020,** *49* (28), 9632-9640.
S4. Rigaku Oxford Diffraction *CrysAlis^{Pro} Software system*, 1.171.41; Rigaku Corporation: 2021.
S5. Sheldrick, G. M., *SHELXT* - Integrated space-group and crystal-structure determination. *Acta Cryst.* **2015,** *A71* (1), 3-8.
S6. Sheldrick, G. M., Crystal structure refinement with *SHELXL*. *Acta Cryst.* **2015,** *C71*, 3-8.
S7. Dolomanov, O. V.; Bourhis, L. J.; Gildea, R. J.; Howard, J. A. K.; Puschmann, H., *OLEX2*: a complete structure solution, refinement and analysis program. *J. Appl. Cryst.* **2009,** *42*, 339-341.
S8. Macrae, C. F.; Sovago, L.; Cottrell, S. J.; Galek, P. T. A.; McCabe, P.; Pidcock, E.; Platings, M.; Shields, G. P.; Stevens, J. S.; Towler, M.; Wood, P. A., *Mercury 4.0*: from visualization to analysis, design and prediction. *J. Appl. Cryst.* **2020,** *53*, 226-235.
S9. Sarigul, N.; Korkmaz, F.; Kurultak, I., A New Artificial Urine Protocol to Better Imitate Human Urine. *Sci. Rep.* **2019,** *9* (1), 20159.
S10. Saadati, N.; Abdullah, M. P.; Zakaria, Z.; Sany, S. B.; Rezayi, M.; Hassonizadeh, H., Limit of detection and limit of quantification development procedures for organochlorine pesticides analysis in water and sediment matrices. *Chem. Cent. J.* **2013,** *7* (1), 63.
S11. Wang, H. L.; Zhou, G. D.; Chen, X. Q., An iminofluorescein-Cu2+ ensemble probe for selective detection of thiols. *Sens. Actuator B. Chem.* **2013,** *176*, 698-703.
